# Targeting PTB for Glia-to-Neuron Reprogramming In Vitro and In Vivo for Therapeutic Development in Neurological Diseases

**DOI:** 10.3390/biomedicines10020399

**Published:** 2022-02-07

**Authors:** Matilde Contardo, Roberta De Gioia, Delia Gagliardi, Giacomo Pietro Comi, Linda Ottoboni, Monica Nizzardo, Stefania Corti

**Affiliations:** 1 Dino Ferrari Centre, Department of Pathophysiology and Transplantation, University of Milan, 20122 Milan, Italy; matilde.contardo@studenti.unimi.it (M.C.); delia.gagliardi@unimi.it (D.G.); giacomo.comi@unimi.it (G.P.C.); 2 Neurology Unit, Foundation IRCCS Ca’ Granda Ospedale Maggiore Policlinico, 20122 Milan, Italy; roberta.degioia@policlinico.mi.it (R.D.G.); linda.ottoboni@policlinico.mi.it (L.O.); monica.nizzardo@policlinico.mi.it (M.N.)

**Keywords:** PTB, reprogramming, neuron, neurodegenerative diseases

## Abstract

In vivo cell reprogramming of glial cells offers a promising way to generate new neurons in the adult mammalian nervous system. This approach might compensate for neuronal loss occurring in neurological disorders, but clinically viable tools are needed to advance this strategy from bench to bedside. Recently published work has described the successful neuronal conversion of glial cells through the repression of a single gene, polypyrimidine tract-binding protein 1 (*Ptbp1*), which encodes a key RNA-binding protein. Newly converted neurons not only express correct markers but they also functionally integrate into endogenous brain circuits and modify disease symptoms in in vivo models of neurodegenerative diseases. However, doubts about the nature of “converted” neurons, in particular in vivo, have been raised, based on concerns about tracking reporter genes in converted cells. More robust lineage tracing is needed to draw definitive conclusions about the reliability of this strategy. In vivo reprogramming and the possibility of implementing it with approaches that could be translated into the clinic with antisense oligonucleotides targeting a single gene like *Ptbp1* are hot topics. They warrant further investigation with stringent methods and criteria of evaluation for the ultimate treatment of neurological diseases.

## 1. Introduction

Neurodegenerative diseases are disabling and often fatal disorders characterized by the progressive loss of specific neuronal subpopulations in various parts of the nervous system and thus specific profiles of neurological dysfunction.

Neurons in the human central nervous system (CNS) are not normally replaced through adult neurogenesis once they are lost, aside from a negligible fraction [1,2,3]. Thus, methods to promote the generation of new neural cells in the adult mammalian brain have been intensively investigated during the past decades [4]. Three main approaches to produce new neurons in the adult brain have been explored: (1) cell transplantation of exogenous neuronal cells/precursors [3,5,6], (2) activation of the endogenous neurogenic capacity of neuronal progenitors in specific zones [7], and (3) reprogramming (or direct conversion or transdifferentiation) of non-neuronal cells, conventionally of abundant glial cells into neurons [8,9,10,11]. 

The strategy of direct neuronal conversion is based on the combinatorial expression of lineage-specific neural transcription factors (TFs) that can turn fibroblasts or glial cells into neurons In Vitro, and likely also in vivo, without passage through a stem cell state [12]. While the transfer and temporally correct expression of different TFs can be complex, the silencing of a single gene can be more straightforward.

Recently, different groups have reported the successful direct conversion of fibroblasts and glia into neurons by reducing the expression of a single target, polypyrimidine tract-binding protein 1 (PTBP1, alias PTB) [13,14]. PTB was initially identified as a polypyrimidine tract-binding protein able to bind to heterogeneous nuclear RNA, placing it in a family of RNA-binding proteins (hnRNP I) [15,16]. The PTB family comprises three alternatively spliced isoforms in mammals: PTBP1 (PTB) expressed in most cell types; PTBP2, also known as neural PTB (nPTB) or brain PTB (brPTB), exclusively present in the nervous system; and PTBP3, also known as ROD1, which is predominantly expressed in immune cells [17]. 

Although the mechanism of PTB activity is not completely understood, it is well known that PTB has many roles in the cell [18,19]: in nuclei, PTB is mostly involved in pre-mRNA splicing events [20], while, in the cytoplasm it is implicated in internal ribosome entry site (IRES)-mediated translation initiation of many different genes, including insulin [21], *p53* [22], and circadian clock gene *Period1* [23]. Furthermore, PTB has a key role in RNA stability, post-translational modifications, transport, and metabolism [24,25]. Another relevant function is its involvement in cell type differentiation [26,27] and cancer [19].

PTB plays a key role in neuronal induction. Thanks to a programmed splicing switch during neural development, it is progressively downregulated to license the expression of nPTB, which is key to neuronal maturation, while the expression of both PTB and nPTB is reduced when neurons mature [17]. Interestingly, while sequential silencing of PTB and nPTB is required to convert human fibroblasts into functional neurons [28], in mice the silencing of PTB alone is sufficient to efficiently convert fibroblasts into functional neurons [17]. 

**Figure 1:**
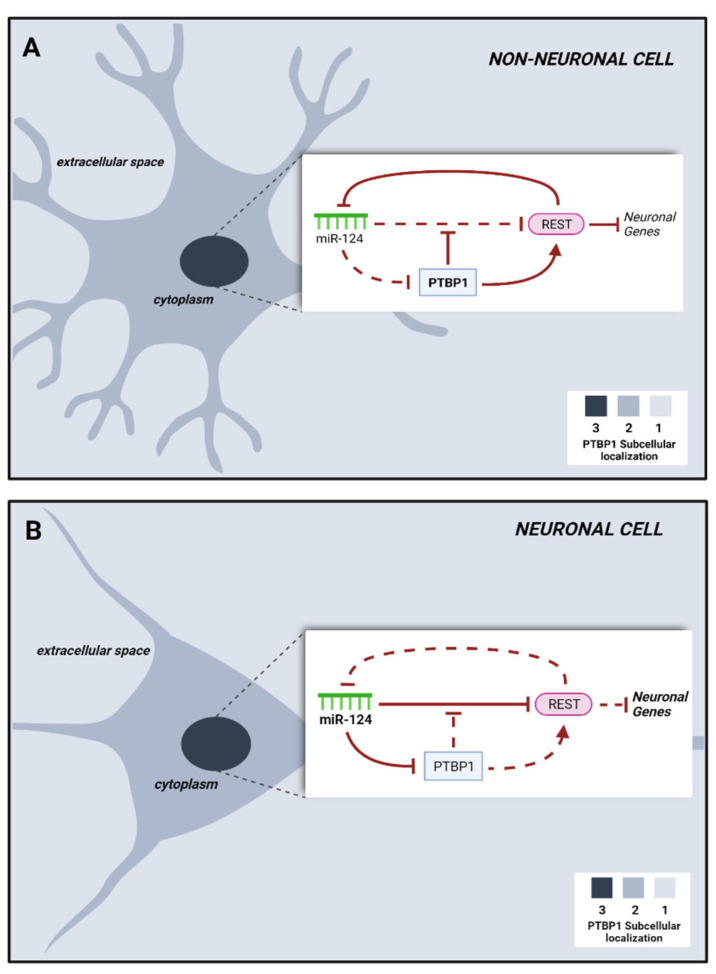
Schematic representation of PTB regulation in mouse non-neuronal (**A**) and neuronal cells (**B**) as described in [17]. In non-neuronal cells, the expression of PTB guarantees the activity of the REST complex in silencing neuronal genes. In neuronal cells, miR-124 upregulation blocks both PTB and the REST complex, allowing the expression of neuronal genes.

Briefly, in fibroblasts PTB acts by blocking a neuronal induction loop in which the microRNA 124 (miR-124) dismantles the transcriptional repressor REST, which conventionally silences a variety of neuronal genes, including miR-124. Suppression of PTB allows miR-124 to silence REST, thus licensing the expression of neuron-specific TFs involved in neurogenesis and of miR-124 itself, whose expression further blocks PTB and sustains the neurogenic loop [28] (Figure 1). Silencing of PTB promotes the expression of nPTB, which blocks the transcriptional activator of neuronal genes *BRN2* and is blocked by miR-9. Both *BRN2* and miR-9 are important for neuronal maturation. 

As previously mentioned, the in vitro silencing of PTB in mice and in humans promotes the expression of nPTB, but the expression levels differ. In humans nPTB is constantly expressed and its silencing is required for complete neuronal maturation, while in mice this sequential silencing is not necessary, due to the transient nature of nPtb upregulation [17,26,28,29]. 

In general, non-neuronal cells such as fibroblasts can be efficiently transdifferentiated toward a neuronal phenotype through interference with the PTB/nPTB pathway. In vivo approaches have found a suitable, reprogrammable, non-neuronal cell type in glial cells. Glial cells are abundant, proliferate upon injury, and seem highly plastic. 

Here, we summarize recent evidences for the reprogramming of fibro-glia to neurons through PTB silencing and discuss current controversy regarding this work and the therapeutic potential approach in neurological disease, adopting a comprehensive view comparing all the techniques applied both in In Vitro and in vivo models, to better clarify possible future fields of investigation.

## 2. PTB Silencing Approaches

PTB pathway is summarized in Table 1.

### 2.1. Interference with PTB-Regulated microRNA Circuits Turns Non-Neuronal Mammalian Cells into Neurons 

In 2013, Xue and colleagues [28] presented evidence that downregulation of *PTB* can promote a neuronal phenotype in non-neuronal cell lines because it interferes both with PTB-dependent alternative splicing and with microRNA circuits involved in the REST complex. The transdifferentiation to neurons was tested in HeLa cells, human embryonic carcinoma stem cells (NT2), mouse neural progenitor cells (N2A), human retinal epithelial cells (ARPE19), and primary mouse embryonic fibroblasts (MEFs) treated with short hairpin RNA (shRNA). The cells were analyzed for neurite outgrowth, expression of major neuronal markers such as beta III-tubulin and MAP2, and synaptic currents. Most cell lines, 5 days to 2 weeks after *PTB* knockdown, in appropriate medium, activated a neuralization process.

RNA-seq analyses and quantitative RT-PCR on PTB-depleted MEF cells were instrumental in identifying a spectrum of up- or downregulated genes involved in neuronal differentiation, such as the TFs *Ascl1*, *Brn2*, and *Myt1l* [30], as well as *NeuroD1*, which is known to enhance neurogenesis in human fibroblasts [31], and miR-124 and miR-9, which sustain TFs during the neurogenic process [32]. 

In particular, the data showed a connection between PTB and the REST complex, which was already known to repress a large set of neuronal genes in non-neuronal cells [33] and of which miR-124 is a part. Modulation of specific microRNAs by PTB downregulation alters various components of the REST complex, including SCP1 and CoREST, thus inhibiting the action of the complex and collectively promoting the expression of specific neuronal genes in non-neuronal cells. Furthermore, PTB acted as a regulator of RNA stability by interacting with the 3’ UTR RNA secondary structure of many genes, probably along with microRNAs. PTB downregulation resulted in the activation of the miR-124/REST loop, of which PTB is a target but also a potent negative regulator of both miR-124 and other micro-RNAs.

### 2.2. Neuronal Reprogramming of Human Cells Is Mediated by the Sequential Activation of Two Key Gatekeepers

Given the reproducibility of the PTB/miR-124/REST loop induced by *PTB* knockdown and the high conservation of this pathway in mammals, Xue and colleagues further explored a molecular strategy to induce reprogramming in two lines of human adult fibroblasts (HAFs) [34].

*PTB* downregulation alone, with the administration of small molecules, resulted in a highly efficient conversion of HAFs into TuJ1+ cells. However, the converted neuron-like cells presented little expression of markers of mature neurons (MAP2, neurofilament-NF, neuronal nuclear antigen-NeuN) and failed to show neuronal activity, suggesting that the conversion process stopped prematurely. Indeed, in contrast to what was previously observed in MEFs [28], RNA sequencing in HAF-derived neuronal cells showed persistent expression of the neural RNA-binding protein PTBP2 (nPTB) indicating the presence of a human-specific barrier to complete neuronal maturation. Notably, nPTB depletion, induced after *PTB* knockdown, was able to generate MAP2-positive neuron-like cells, which survived in co-culture with glial cells for 3 months. Further, sequential depletion of PTB and nPTB induced the expression of *BRN2*, a cortex-specific transcription factor, which in turn induced the expression of markers of mature neurons, including MAP2, NCAM, vGLUT1, and NeuN. Finally, spontaneous postsynaptic activities were detected after co-culturing HAF-derived neuron-like cells with glial cells for 3 to 4 weeks.

The relevance of BRN2 in mediating neuronal maturation was further validated in human neural progenitor cells (hNPCs). In this context, *BRN2* knockdown did not affect early neuronal differentiation but compromised mature neuronal marker expression and the onset of electrical firing.

Thus, BRN2 is required for hNPC differentiation into mature functional neurons. The analysis of downstream processes revealed that BRN2 binds and regulates both miR-124 and miR-9 expression in hNPCs. The former was found to take part in the neuronal conversion process, while the latter was shown to drive neuronal differentiation and to mediate the progression to functional neurons by inducing nPTB repression.

Thus, differently from MEFs, the nPTB-BRN2–miR-9 loop is responsible for the generation and maturation of neuronal cells from human fibroblasts. Specifically, the sequential inactivation of PTB and nPTB triggers *BRN2* overexpression, which in turn induces miR-9; increased miR-9 levels post-transcriptionally further decrease *nPTB* expression, sustaining the loop. It is still unclear which signals trigger this loop in the human brain during development.

These findings suggest the existence of two sequential gatekeepers controlling neuronal conversion and maturation in brain development. Overturning these gatekeepers enables the deterministic reprogramming of human fibroblasts into functional neurons.

### 2.3. Neural Reprogramming of Rat Adult Resident Striatal Oligodendrocytes by an Adeno-Associated Viral Vector

In vivo, one target for this neural reprogramming approach could be the resident oligodendrocyte precursor cells (OPCs) that sustain physiological cell replacement via active proliferation upon injury [35]. 

As an alternative to neuronal reprogramming via complex breeding of transgenic mice or retrovirus-driven gene expression, Weinberg et al. have proposed using a specific miRNA (GFP tagged) capable of silencing PTB protein, which they cloned into an adeno-associated viral vector that encodes an oligotrophic capsid protein [35].

Ten days post-injection of the recombinant AAV4miRNA-GFP virus into the rat striatum, the majority of GFP-positive cells were OLIG2+ oligodendrocytes. After 6 weeks, the constitutive expression of the miRNA-GFP induced the transdifferentiation of transduced oligodendrocytes towards a striatal neuron phenotype, as assessed via morphology and colocalization with NeuN, DARRP32, or parvalbumin.

The pattern of expression was stable even in the long-term, indicating the ability of this construct to be constitutively expressed. 

The functionality of the AAV4miRNA-GFP-induced striatal neurons was validated through patch-clamp recordings of spontaneous action potentials or postsynaptic currents elicited with current injections.

Further, 3 months after AAV4miRNA-GFP administration, confocal microscopy of fluorescent beads infused into the globus pallidus and the substantia nigra of rats and subsequent analysis showed that a number of striatal cell bodies were GFP+, indicating the effective functionality of presynaptic terminals capable of internalizing the fluorescent beads and performing retrograde axonal transport.

Thus, the single AAV4miRNA construct specific for PTB developed by Weinberg et al. [35] has the potential by itself to efficiently replenish the neuronal landscape via transdifferentiation of resident oligodendrocytes into functional neurons. 

### 2.4. Neural Reprogramming of Mouse Retinal and Striatal GFAP+ Cells by CRISPR-CasRx

In vivo neural reprogramming has been further implemented by Zhou et al. [36] on Müller glia (MG) cells using Cas13d (CasRx) CRISPR technology against *Ptb*, to transdifferentiate MGs into retinal ganglion cells (RGCs) as a potential therapeutic approach to restore visual function in retinal degenerative diseases [36]. 

CRISPR-CasRx can efficiently manipulate RNA transcripts with high efficiency and specificity, and CasRx is optimal in vivo due to its small size. Zhou et al. [36] exploited the CRISPR-CasRx mechanism with two gRNAs targeting, respectively, exon IV and exon VII of the *Ptb* mRNA.

In Vitro, *Ptbp1* mRNA levels in neuronal stem cells (N2a) and astrocytes were reduced by 87% ± 0.4% and 76% ± 4%, respectively.

CRISPR-CasRx for *Ptb* was then tested in vivo in Ai9 mice via local AAV9 (Rosa-loxP-TdTomato mouse) subretinal injection. AAV9-*Gfap*-GFP-Cre and AAV9-*Gfap*-CasRx-*Ptbp1* were co-injected to specifically label retinal MG cells with tdTomato and drive the *Ptbp1* knockdown. 

After 1 month, the tdTomato+ cells presented in the retinal ganglion layer of these mice co-immunostained for RGC markers, indicating effective conversion from MG to RGC in the mature retina.

Further, to explore the functionality of converted RGCs, the researchers induced a retinal injury through intravitreal NMDA injections. At 2 to 3 months after the induced chemical toxicity, mice were treated with the AAV9 constructs (Cas-*Ptb+*). One month later, the number of viable RGCs was significantly increased compared with that in the control mice (injured and without *Ptp* downregulation). Reprogrammed RGCs extended tdTomato+ axonal projections to the central target area of the dorsal lateral geniculate nucleus and to the superior colliculus of the brain, confirming the correct generation of effectively functional RGCs. Moreover, light stimulus-evoked visual responses were recorded, suggesting that MG conversion could partially restore visual functions in mice.

The same reprogramming gene therapy approach was tested in the striatum of a Parkinson’s disease (PD) mouse model to restore dopamine expression and mitigate motor dysfunction associated with PD. 6-OHDA-induced PD mice were given injections of AAV9-*Gfap*-mCherry and CasRx-*Ptbp1* constructs in the ipsilateral striatum. Knockdown of *Ptbp1* resulted in an increasing number of cells expressing tyrosine hydroxylase (TH) over the following months in PD mice, and the majority of TH+ cells were mCherry+, suggesting that mainly an astrocyte-to-neuron conversion occurred. Further, most of the mCherry+TH+ cells also expressed the marker of mature dopaminergic neurons, dopamine transporter (DAT), suggesting that not only a correct astrocyte-to-neuron conversion but also effective induction towards a dopaminergic phenotype had occurred. Patch-clamp analysis confirmed the neurophysiological activity of the transdifferentiated neurons.

Overall behavioral analysis of motor functions revealed that symptoms of Parkinson’s disease were alleviated in treated mice compared with controls.

### 2.5. In Vitro and In Vivo Neural Reprogramming of Astrocytes by AAV PTB Silencing in Mice

Almost back-to-back to the work of Zhou et al. [36], evidence of the possibility of converting astrocytes into neurons was independently described by the Fu laboratory [13]. Indeed, leveraging the consolidated evidence that PTB, nPTB, REST, miR-124, miR-9, and BRN2 are key players in the establishment of cell identity in fibroblasts, astrocytes, and neurons, with feed-forward inhibitory loops, Qian and colleagues expanded their original work of fibroblast-to-neuron conversion [34], via sequential *Ptb* downregulation, to the context of astrocytes, which express an RNA program intermediate between fibroblasts and neurons [13]. The authors first delivered, In Vitro, an RNA silencing hairpin (shRNA) as a lentivirus to reduce *Ptb* expression in mouse cortical astrocytes. In 4 weeks, the cells gained neuronal morphology and expression of Tuj1 and MAP2 and acquired a brain region-specific gene expression profile: for example, midbrain astrocytes gave rise to dopaminergic neurons and manifested both induced and spontaneous firing. Moving to an in vivo experiment, the authors transduced the striatum of a *Gfap*-Cre transgenic mouse using an adeno-associated virus serotype 2 (AAV2) engineered to encode shRNA against *Ptb* linked to a loxP-RFP reporter that could be expressed when infection occurs in cells expressing Cre-recombinase under the astrocyte-specific *Gfap* promoter. This tracing strategy is similar to the one used with AAV9 by Zhou et al. [36]. When the empty vector was used in vivo, 10 weeks after injection in the midbrain no conversion was detected, while 3 weeks after injection 20% of RFP cells were NeuN+. By week 10, this reached 80%, with expression of synaptic markers. By week 12, RFP-NeuN+ neurons also expressed TH, a marker of mature dopaminergic neurons, and manifested mature electrophysiological properties with time-dependent integration into the nigro-striatal network.

When silencing was induced in the striatum and in the cortex, although the percentage of NeuN+ cells was similar to that in the midbrain region, the frequency of dopaminergic neurons was higher in the midbrain while reprogrammed neurons in the cortex were either CTIP2- or CUX1-positive neurons, suggesting that astrocytes have regional specificity and can give rise to different types of neurons. The same was true in culture, although the yield of midbrain astrocyte conversion into dopaminergic neurons was 35% in vivo and 10% In Vitro, likely as a consequence of environmental cues that contribute to the astrocyte transition in vivo. 

Newly trans-differentiated TH+ neurons in the striatum developed long axons able to reach target sites of connectivity, such as the caudate putamen or nucleus accumbens, and displayed mature synaptic features. The potential of the Ptb-dependent regenerative strategy was also tested in chemically induced PD [37]. One month after toxic administration in the medial forebrain bundle, only 10% of TH+ neurons were spared, while astrogliosis was present. However, from 2.5 to 3 months after *Ptb* silencing via AAV, administered one-month post toxic injury, new TH+ RFP neurons arose. This therapeutic approach promotes the transdifferentiation of a number of cells corresponding to around 20% of the original neurons in the healthy animal. Thus, the final total fraction of functional neurons is one-third of the original number, with a moderate increase in fiber density. The restored network’s dopamine level reached up to 65% of the basal level, and within 3 months, disease-relevant motor phenotypes such as time-dependent and progressive forelimb use were rescued. Motor improvement occurred in both young and old animals, although in the latter it was not complete, suggesting an age-dependent decrease of neuronal reprogramming efficiency.

Of note, the authors were able to ascertain in a very elegant way that transdifferentiated neurons are actually responsible for the phenotypic recovery. Upon injury, mice were treated with an AAV engineered with both *sh-PTB* and a flox-embedded inhibitory variant of the human muscarinic receptor (DREADD) [38] to both induce reprogramming and express a receptor that can trigger hyperpolarization. Indeed, when clozapine-N-oxide (CNO) was administered to PD mice treated with the viral construct, the beneficial effect of the transdifferentiated cells was abrogated and transient signs of PD disorder returned 40 minutes after CNO intraperitoneal injection. This did not occur when the AAV vector lacked DREADD or when CNO was administered to healthy mice. 

### 2.6. In Vitro and In Vivo Neural Reprogramming of Astrocytes by Antisense Oligonucleotide (ASO) PTB Silencing 

As an alternative to shRNA lenti- or adeno-associated mediated viral strategy to reduce *Ptb* mRNA, antisense oligonucleotides (ASO) injected into the cerebral spinal fluid (CSF) can achieve similar results.

Although Qian and colleagues [13] mainly focused their work on neuronal reprogramming of astrocytes via AAV *Ptb* silencing, they also tested astrocyte transdifferentiation using a specific ASO as a feasible, single-step approach to treat Parkinson’s disease and likely other neurodegenerative conditions. Indeed, Qian and colleagues [13] were the first to successfully implement the transdifferentiation of astrocytes into neurons, both in 2D culture and in transgenic mice using a specific ASO. 

This attempt was followed by a larger screening of 200 ASOs on the 4T1 mouse cell line, which led to the identification of two top candidates able to reduce *Ptb* expression [14]. The most promising ASO candidate showed molecular efficacy In Vitro on mouse astrocytes and human brain organoids from control subjects and in vivo with minimal animal toxicity, in healthy mice. Downregulation of *PTB* in organoids correlated with increased numbers of NeuN+/MAP2+ cells and reduced numbers of GFAP+ cells, *bona fide* neurons, and astroglia. In vivo, in 3-month-old mice, a single intracerebroventricular (ICV) ASO injection downregulated *Ptbp1* 3 days post treatment and increased *nPtb* 15 days post treatment. At 2 months post treatment, there was an increase of ~1% of new NeuN+ cells with neuronal morphology similar to endogenous neurons in the I and IV layers of the cortex and in the CA1 region of the hippocampus. The authors leveraged a congenic mouse carrying an inducible Cre transgene under the human *GFAP* promoter [39] and a chicken β-actin promoter (CAG)-LSL-tdTomato domain to trace cells and ascertain whether GFAP-expressing cells transdifferentiated into neurons. In the granule cell layer of the dentate gyrus of the hippocampus, where radial glial cells, stem cell precursors, and astrocytes express GFAP and where neurogenesis takes place, a single ASO injection induced up to 7.8% new transdifferentiated neurons. Remarkably, PTB-ASO injection was also able to promote up to 5% conversion of GFAP+ cells into NeuN+ neurons in the aged brains of 12-month-old mice, while only a few new neurons appeared in the cortex. Newly generated hippocampal neurons displayed canonical neuronal development, morphology, markers, and neurophysiological properties. 

Interestingly, based on Tomato expression, no astrocyte depletion could be observed in the hippocampus, where transdifferentiation was robust, likely because PTB-ASO treatment also triggered, in both mice and organoids, proliferation (Ki67+ cells) of radial glia-like GFAP+ precursors, in line with existing understanding of homeostatic mechanisms of astroglia [40]. Of note, as they began their conversion, tdTomato+ cells exhibited morphological characteristics of radial glial cells, with triangular-shaped DCX+ cell bodies and GFAP+ dendrites, as an intermediate cell type. Over a 2-month period, these cells could functionally integrate and influence the behavioral response of the mice. They extended their dendrites into the perforant path and their axons into CA3, sending action potentials that modified hippocampus-dependent mouse behavior and receiving inhibitory and excitatory signals.

Maimon and colleagues [14], with this mouse model, showed that PTB-ASO promoted the transdifferentiation of radial glia or astrocytes into neurons, and they also reproduced the PTB-ASO therapeutic approach in human organoids. Moreover, in non-engineered mice the PTB-ASO treatment was also clearly able to promote the formation of new immature (DCX+) neurons, which correlated with improved memory as assessed using the Barnes maze test. 

Of note, the converted neurons acquired morphologies matching the characteristics of the brain region where they arose, highlighting that the particular remodulation of gene expression primed by the pharmacological intervention is a function of the regional specificity of the glia. Detailed gene expression monitoring and proteomic characterization are now needed to identify and harness glial cell-intrinsic or environmental factors.

ASO technology has been deeply studied and tested in the last 15 years and successfully taken to the market for the fatal childhood disease spinal muscular atrophy [41]. It is in early and late clinical trial development for other neurodegenerative disorders such as amyotrophic lateral sclerosis [42] and Parkinson’s and Alzheimer’s diseases [43]. Progress has been made in generating induced neurons (iNs) using ASOs [44]. There is also evidence that ASOs can be used to tackle neurodegenerative disorders as well as rejuvenate the brain after age-related progressive neuronal loss via transient PTB suppression to facilitate neuronal replacement. Despite these findings, most proposed strategies are not ready for clinical testing [45].

Optimization of ASO chemistry to improve PTB suppression, which is less than 50% in most brain regions, is required because treatment with optimized ASOs can achieve 95% suppression [46,47,48]. In the hippocampus, PTB expression was reduced by more than 50% and glia-to-neuron conversion was also robust in aged mice, suggesting that at least a 50% reduction is needed to trigger efficient conversion. 

### 2.7. Astrocyte Conversion Is Not the Only Hypothesis

Despite the papers claiming that ectopic expression or knockdown of certain factors leads to endogenous astrocyte conversion into new neurons, a recently published paper questions this hypothesis [49]. Indeed, using different, stringent, lineage-mapping strategies in the mouse brain, the authors showed that the cells positive for neuronal markers identified after the use of the two most used conversion factors, NEUROD1 and PTB, were not derived from astrocytes. In particular, by tracing YFP-expressing astrocytes and using DCX and BrdU, they elegantly demonstrated that co-expression of AAV-mediated *NEUROD1* or *shPTB* with a GFP reporter did not provide evidence of astrocyte-to-neuron conversion. Specifically, despite the numerous neurons observed after putative conversion, the overall neuronal density remains unchanged, which contrasts with the net increase in neurons expected after robust astrocyte conversion. The explanatory hypothesis was that the GFAP promoter cell specificity can be altered by trans or cis mechanisms of downstream factors or expressed genes induced by *NEUROD1* or *shPTB* and thereby mislead observers as to the origin of the detected neurons. To support those conclusions and to pinpoint the alternative source of the newly originated neurons that improved the phenotype of traumatic brain injury in mice, the authors used a retrograde labeling approach. This unveiled the role of endogenous neurons of unclear origin. The authors hypothesized a migration of endogenous neural stem cells or fusion of microglia and endogenous neurons, but stringent genetic lineage tracing is essential to follow fate changes in these cells during conversion and provide a definitive answer to the question.

Moreover, as regards the observed behavioral improvements, *NEUROD1* and *shPTB* can have an impact independent of astrocyte conversion, because of their respective intrinsic neurogenic activity [50,51] and ability to decrease neuronal vulnerability [52].

## 3. Conclusions 

The discovery that PTB reduction in glial cells can reprogram them into neurons began from the seminal observation of this phenomenon in murine and then human fibroblasts, followed by the investigation of the transdifferentiation mechanisms via a PTB-miRNA modulated loop [17], and then more recently with the exploration of in vivo conversion in rodent disease models with the final aim of clinical translation for the treatment of neurological diseases. Nevertheless, different technical and biological difficulties need to be solved before a successful transfer from bench to bedside. 

The evolutionary differences between human and mouse are reflected in the different regulatory mechanisms of PTB/nPTB-mediated loops [17], and undoubtedly, translation to humans is still limited by the moderate efficiency of reprogramming, by possible cellular mistargeting, and by potential side effects caused by local astrocyte depletion. This represents a major limitation in terms of translatability, and to date, most of the existing literature focuses on mouse-based research. 

Since aging is the primary risk factor for most neurodegenerative diseases, including Alzheimer’s and Parkinson’s disease, the possibility of modulating neural replacement in this context is currently a hot topic [53] that needs to be further investigated and also for nerve damage caused by injures or even for blindness [54]. Nonetheless, how aging can impact the feasibility of glia-to-neuron reprogramming is still largely unknown, although the Cleveland group paper [14] demonstrated that PTB reduction benefits glia-to-neuron conversion in aged mouse brains as well. The authors proposed the hypothesis that reducing the PTB-stimulated proliferation of radial glia-like cells triggered their conversion into new granule neurons in the aged rodent brain through what appears to be the typical process occurring in the hippocampus during development and in young adults.

If new neurons originate from astrocytes, astrocyte depletion should be expected. This could be beneficial in some disorders with pronounced astrocytosis but also detrimental if excessive, given the important functions of glia in the CNS. This risk was apparently ruled out in the work of Maimon et al. [14], where actual conversion into new neurons was obtained from radial/precursor glial cells rather than mature astrocytes. However, this evidence should be confirmed in depth in further analyses.

As regards the different tools that have been implemented to downregulate PTB, its permanent downregulation with an AAV-shRNA plasmid can bring potential risks, while the use of ASOs can overcome this problem, since it can be suspended, representing a more clinically amenable therapeutic approach. However, optimizing ASOs for minimal toxicity and maximal efficacy is still important.

Further, it is intriguing that the morphology of the newly reprogrammed neurons seems to be dictated by the brain region in which they originated, matching the respective characteristics of granule, pyramidal, or cortical neurons. Moreover, in some regions, like the hippocampus or midbrain, the conversion rate is more efficient, probably due to the basal levels of PTB and nPTB, as suggested also in the computational predictive model from Merlevede et al. [55]. 

One hypothesis is that neuronal identity is determined both by local signals present within each specific brain region and by the intrinsic gene expression profile of the glial cells. Further studies are needed to identify the key elements (glial cell-intrinsic or environmental extrinsic signals) that govern this transition. On the other hand, other authors suggest that the perfect match of the neuronal subtype product with the brain area could be the result of genetic artifacts in cell tracing. Nonetheless, other authors argue that they can demonstrate a longitudinally progressive conversion of intermediate glial precursors in the hippocampus. The definitive, artifact-free elucidation of the actual rate of conversion is of the utmost importance in the development of this approach.

Overall, further studies are needed to explore the specific mechanisms of glia-to-neuron conversion via PTB silencing and to understand whether, in the long run, the findings of these studies will show good potential for the treatment of neurological diseases. The efficacy of PTB modulation as a therapeutic strategy is still in a very preliminary stage, and it is necessary to still take advantage of murine studies. To date, few works in the literature focus only on human-based models since PTB modulation is easier in mice both in In Vitro and in vivo models. Once the real therapeutic value of this pathway is established, it will be necessary to translate it for a clinical approach, basing further studies in humans, due to the already known great differences in regulatory mechanisms between human and mice. 

## Figures and Tables

**Table 1:** Summary of PTB-silencing approaches reported in the review. NT2 = human embryonic carcinoma stem cells; N2A = mouse neural progenitor cells; ARPE19 = human retinal epithelial cells; MEFs = primary mouse embryonic fibroblasts; HAFs = human adult fibroblasts; OPCs = oligodendrocyte precursor cells; MGCs = Müller glia cells; RGCs = retinal ganglion cells.

Approach-tools	Strategy	Cell Source	Outcome	Reference
lenti-shRNA	In vitro	HeLa, NT2, N2A, ARPE19, MEF	Neurons	Xue et al., 2013 [28]
lenti-shRNA	In vitro	HAFs	Mature neurons	Xue et al., 2016 [34]
AAV4miR-GFP	In vivo	Rat OPCs	Striatal neurons	Weinberg et al., 2017 [35]
CRISPR-CasRx	In vivo	Mouse MGCs	RGCs	Zhou et al., 2020 [36]
CRISPR-CasRx	In vivo	Mouse striatum astrocytes	Dopaminergic neurons	Qian et al., 2020 [13]
lenti-shRNA	In vitro	Mouse cortical astrocytes	Dopaminergic neurons	
AAV2-shRNA	In vivo	Mouse cortical astrocytes	Dopaminergic neurons	
ASO	In vitro	Mouse astrocytes	Neurons	Maimon et al., 2021 [14]
ASO	In vitro	Astrocytes in human brain organoids	Neurons	
ASO	In vivo	Mouse glial cells	Neurons	
CRISPR-CasRx/AAV5-shRNA	In vivo	Neurons	Neurons	Wang et al., 2021 [49]

## Data Availability

Not applicable.
